# Pattern analysis of 532- and 1,064-nm picosecond-domain laser-induced immediate tissue reactions in *ex vivo* pigmented micropig skin

**DOI:** 10.1038/s41598-019-41021-7

**Published:** 2019-03-12

**Authors:** Hee Chul Lee, James Childs, Hye Jin Chung, Jinyoung Park, Jumi Hong, Sung Bin Cho

**Affiliations:** 1 R&D Center, Lutronic Corporation, Goyang, Korea; 2 Global Center, Lutronic Corporation, Billerica, MA USA; 3 Department of Dermatology, Boston University School of Medicine, Boston, MA USA; 4 Department of Dermatology and Cutaneous Biology Research Center, International St. Mary’s Hospital, Catholic Kwandong University College of Medicine, Incheon, Korea; 5 Yonsei Seran Dermatology and Laser Clinic, Seoul, Korea

**Keywords:** Structural biology, Cells

## Abstract

Optical pulses from picosecond lasers can be delivered to the skin as single, flat-top beams or fractionated beams using a beam splitter or microlens array (MLA). In this study, picosecond neodymium:yttrium aluminum garnet laser treatment using a single flat-top beam and an MLA-type beam at the wavelengths of 532 nm and 1,064 nm were delivered on *ex vivo* genotype-regulated, pigmented micropig skin. Skin specimens were obtained immediately after treatment and microscopically analyzed. Single flat-top beam treatment at a wavelength of 532 nm and a fluence of 0.05-J/cm^2^ reduced melanin pigments in epidermal keratinocytes and melanocytes, compared to untreated controls. Additionally, 0.1 J/cm^2^- and 1.3 J/cm^2^-fluenced laser treatment at 532 nm elicited noticeable vacuolation of keratinocytes and melanocytes within all epidermal layers. Single flat-top beam picosecond laser treatment at a wavelength of 1,064 nm and a fluence of 0.18 J/cm^2^ also reduced melanin pigments in keratinocytes and melanocytes. Treatment at 1,064-nm and fluences of 1.4 J/cm^2^ and 2.8 J/cm^2^ generated increasing degrees of vacuolated keratinocytes and melanocytes. Meanwhile, 532- and 1,064-nm MLA-type, picosecond laser treatment elicited fractionated zones of laser-induced micro-vacuolization in the epidermis and dermis. Therein, the sizes and degrees of tissue reactions differed according to wavelength, fluence, and distance between the microlens and skin.

## Introduction

Picosecond-domain lasers target chromophores by irradiating the skin at higher peak power and an extremely shorter pulse duration, compared to nanosecond-domain lasers^1^. Laser treatment therewith provides more effective energy transfer to target chromophores of smaller size than nanosecond-domain laser treatment^2,3^. Furthermore, to generate permanent zones of tissue breakdown, picosecond laser treatment elicits expansion and collapse of great magnitude that is initiated by the irradiation of pigment chromophores in target tissue^3^. Thereby, compared to nanosecond laser treatments, picosecond laser treatment better disintegrates pigment particles into smaller particles that are effectively dispersed into peripheral areas^3^.

Optical pulses from picosecond lasers can be delivered to the skin as single, flat-top beams or fractionated laser beams^4,5^. For the latter, diffractive beam splitters or microlens arrays (MLAs) have been adopted to generate multiple focused beamlets^4^. Therewith, high-intensity, micro-injury zones of laser-induced breakdown are generated in the epidermis or dermis, with limited photothermal injury to the surrounding tissue^4–6^. These laser-induced micro-injury zones have been suggested to stimulate new collagen, mucin, and elastin production in the dermis during the wound repair process to improve the appearance of wrinkles and atrophic scars^5,7^. A previous multiphoton microscopy study provided laser scanning microscopic images of *in vivo* human skin treated by 532-nm and 1,064-nm picosecond neodymium:yttrium-aluminum-garnet (Nd:YAG) lasers using a holographic diffractive beam splitter, and suggested pigment chromophores as the main absorber for initiating the generation of laser-induced tissue breakdown^4^.

In this observational descriptive study, we evaluated the patterns of immediate tissue reactions induced by 532- and 1,064-nm picosecond laser treatment in *ex vivo* genotype-regulated, pigmented micropig skin. To do so, two- and three-dimensional characterization of focused areas, which were generated by delivering MLA-type beams from a picosecond laser, were evaluated. Then, picosecond-domain laser treatments at the wavelengths of 532 and 1,064 nm were performed on the *ex vivo* tissue samples of a pigmented micropig model using a single flat-top beam and an MLA-type beam. Specimens were histologically evaluated following hematoxylin and eosin staining. Furthermore, we also compared the patterns of tissue reactions between nanosecond and picosecond Nd:YAG lasers using a single flat-top beam.

## Results

### Flat-top beam 532-nm picosecond laser-induced tissue reactions

**Figure 1:**
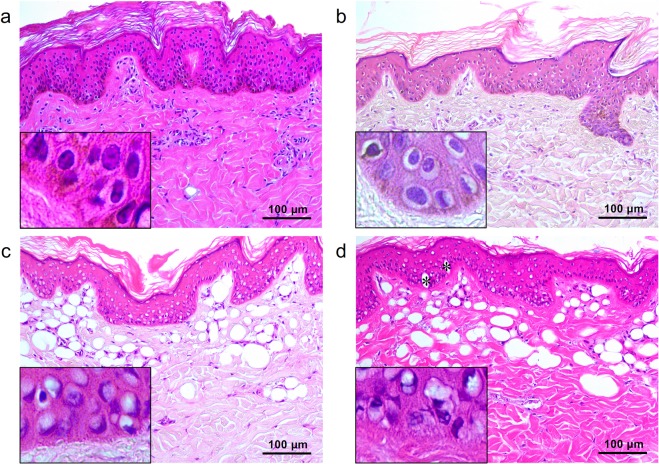
Tissue reactions after 532-nm single flat-top beam picosecond-domain laser treatment. (**a**) Untreated *ex vivo* pigmented micropig skin presented darkly and homogeneously pigmented keratinocytes and melanocytes in the epidermis and collagen fibers, fibroblasts, and dermal microvascular component in the upper dermis. Single pulses of single flat beam, 532-nm picosecond laser treatment at a spot size of 5.3 mm and at the fluences of (**b**) 0.05 J/cm^2^, (**c**) 0.1 J/cm^2^, and (**d**) 1.3 J/cm^2^. Inlets depict close-up views of the basilar keratinocytes, melanocytes, and basement membrane. (**d**) Asterisks indicate giant epidermal vacuoles. H&E stain, original magnification x200.

The untreated *ex vivo* pigmented micropig skin in our study exhibited the characteristic layers of the epidermis, including the stratum corneum in a basket-weave pattern, the stratum spinosum with pigmented, polyhedral keratinocytes, the stratum basalis with darkly and homogeneously pigmented keratinocytes and melanocytes, and an intact basement membrane (BM) (Fig. 1a). The upper dermis was composed of homogenous collagen fibers, fibroblasts, and dermal microvascular components (DMVCs). Immediately after a single pulse of picosecond laser treatment at 532 nm, a 5.3-mm spot size, and a 0.05-J/cm^2^ fluence, melanin pigments in the basal layer were slightly decreased, compared to untreated micropig skin (Fig. 1b). Most of the epidermal keratinocytes presented perinuclear vacuolization. The integrity and thickness of the BM were intact, and DMVCs were mildly dilated.

The 532-nm 0.1 J/cm^2^-fluenced picosecond laser treatment elicited marked vacuolation of keratinocytes and melanocytes throughout all epidermal layers, and melanin pigments in the epidermis were reduced (Fig. 1c). Under high power magnification, vacuoles seemed to have pushed nuclei in sporadic directions and appeared with signet ring-like morphology. Numerous round zones of laser-induced tissue injury were found in collagen bundles at the maximum depth of 274.2 ± 39.0 µm and around dilated DMVCs. Meanwhile, 1.3 J/cm^2^-fluenced laser treatment generated more signet ring-like keratinocytes with extensive vacuolization, and the nuclei thereof were more indented (Fig. 1d), compared to the other experimental settings (Fig. 1b,c). Vacuolar changes in the basal layer were remarkably extensive, and giant elliptical vacuoles with a size of 32.5 ± 1.6 µm × 15.7 ± 2.0 µm were also found in the epidermis (Fig. 1d). The maximum depth of round cavities of laser-induced tissue reaction was estimated at 371.8 ± 30.0 µm in the dermis (Fig. 1d). The maximum depth of tissue reactions was significantly correlated with the power density (*R* = 0.765; *P* < 0.001).

### Flat-top beam 1,064-nm picosecond laser-induced tissue reactions

**Figure 2:**
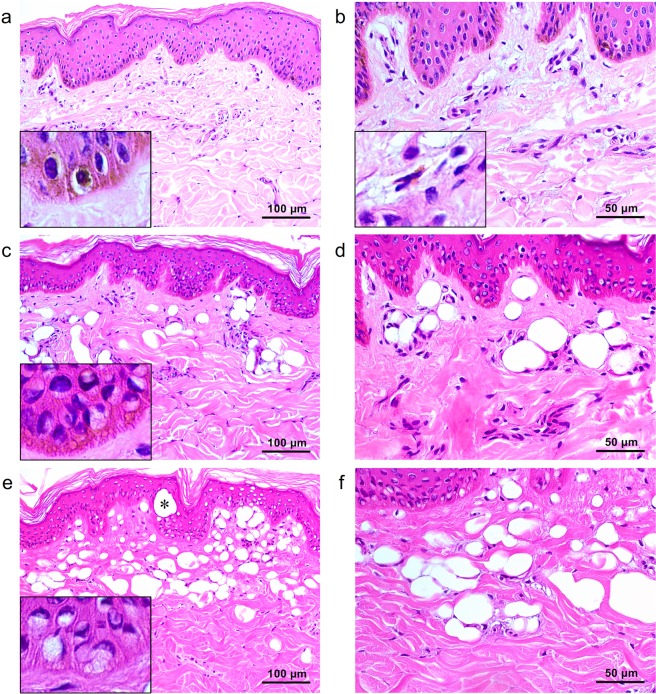
Tissue reactions after 1,064-nm single flat-top beam picosecond-domain laser treatment. Single pulses of single flat-top beam, 1,064-nm picosecond laser treatments at a spot size of 6 mm and the fluences of (**a**,**b**) 0.18 J/cm^2^, (**c**,**d**)1.4 J/cm^2^, and (**e**,**f**) 2.8 J/cm^2^. (**a**,**c**,**e**) Inlets depict close-up views of the basilar keratinocytes, melanocytes, and basement membrane. (**b**) Inlet, close-up view of melanin-containing cell with small, dark-staining nucleus in the dermis. (**d**) Asterisk indicates giant epidermal vacuole. H&E stain, original magnification (**a**,**c**,**e**) x200, (**b**,**d**,**f**) x400.

Single-pulse treatment at 1,064 nm, a 6-mm spot size, and a fluence of 0.18 J/cm^2^ elicited decreased melanin pigments in the basal layer (Fig. 2a), compared to untreated micropig skin. Most of the epidermal keratinocytes exhibited slight perinuclear vacuolization. BM integrity was intact, although clusters of melanin granules or melanin-containing cells were occasionally found in the dermis (Fig. 2b). Meanwhile, 1.4 J/cm^2^-fluence treatment elicited vacuoles of varying sizes in keratinocytes and melanocytes that pushed nuclei in sporadic directions (Fig. 2c). The overall amount of epidermal melanin pigments was markedly reduced; in particular, the melanin particles in the basilar epidermis were homogeneously disintegrated (Fig. 2c,d). Round cavities of laser-induced tissue injury were also found in collagen bundles and around dilated DMVCs at the maximum depth of 212.8 ± 48.7 µm (Fig. 2d).

Skin treated at a fluence of 2.8 J/cm^2^ exhibited signet ring-like keratinocytes with extensive vacuolization of varying size and indented nuclei, compared to the other experimental settings (Fig. 2e). Vacuolar changes in the basilar epidermis were extensive and appeared to involve the BM zone; few or no melanin particles were found. A large oval vacuole (size of 40.0 ± 6.1 µm x 48.9 ± 5.5 µm) was identified in the epidermis (Fig. 2e). DMVCs were mostly dilated and exhibited numerous round cavities of laser-induced tissue injury in collagen bundles (maximum depth, 264.1 ± 44.9 µm), compared to treatment at 1.4 J/cm^2^ (Fig. 2f). Although the depth of vacuolated reactions was slightly deeper at the fluence of 2.8 J/cm^2^, compared to 1.4 J/cm^2^, treatment with 2.8-J/cm^2^ fluence generated more extensive tissue reactions in the more superficial parts of the skin (Fig. 2e,f). Furthermore, the maximum depth of tissue reaction was significantly correlated with the power density (*R* = 0.961; *P* < 0.001). Nonetheless, the differences in the maximum depth of tissue reaction and the size of epidermal giant vacuoles between the wavelengths of 532 nm and 1,064 nm were insignificant (*P* > 0.05).

### Nanosecond-domain Nd:YAG laser-induced tissue reactions

**Figure 3:**
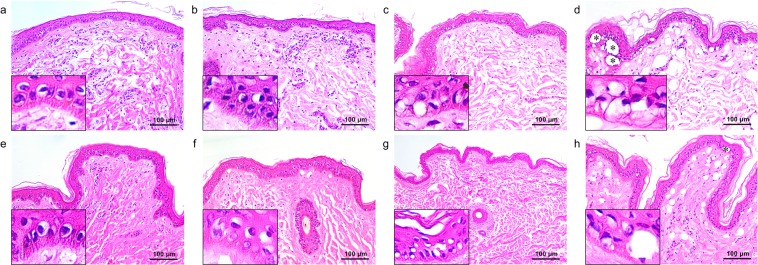
Nanosecond-domain Nd:YAG laser-induced tissue reactions. Single pulses of single flat-top beam, 532-nm nanosecond laser treatment at a spot size of 5.3 mm and the fluences of (**a**) 0.07 J/cm^2^, (**b**) 0.1 J/cm^2^, (**c**) 1.3 J/cm^2^, and (**d**) 1.8 J/cm^2^. Additionally, single pulses of single flat-top beam 1,064-nm picosecond laser treatment at a spot size of 6 mm and the fluences of (**e**) 0.36 J/cm^2^, (**f**) 1.4 J/cm^2^, (**g**) 2.8 J/cm^2^, and (**h**) 4.0 J/cm^2^. Inlets depict close-up views of the basilar keratinocytes, melanocytes, and basement membrane. (**d,h**) Asterisks indicate giant epidermal vacuoles. H&E stain, original magnification x200.

Immediately after single respective pulses of 532-nm nanosecond laser treatment at the fluences of 0.07 J/cm^2^, 0.1 J/cm^2^, and 1.3 J/cm^2^, all skin samples exhibited various degrees of perinuclear vacuolar changes and decreased melanin pigments in the epidermis, without remarkable vacuolar tissue reactions in the dermis (Fig. 3a,c). Giant epidermal vacuoles and marked dermal vacuolar tissue reactions were found at the fluence of 1.8 J/cm^2^ (Fig. 3d). However, dermal tissue reactions were less extensive that those attained with 0.1-J/cm^2^ picosecond laser treatment.

A single pulse of 1,064-nm nanosecond laser treatment at the fluence of 0.36 J/cm^2^ generated mild perinuclear vacuolar changes and decreased melanin pigments, without remarkable vacuolar tissue reactions in the dermis (Fig. 3e). While varying degrees of epidermal vacuoles and mild dermal vacuolar changes were found in the skin treated at the fluences of 1.4 J/cm^2^ and 2.8 J/cm^2^ (Fig. 3f,g), giant epidermal vacuoles and marked dermal vacuolar tissue reactions were found at the fluence setting of 4.0 J/cm^2^ (Fig. 3h). However, dermal tissue reactions generated with 4.0-J/cm^2^ nanosecond laser treatment were less extensive than those obtained with 1.4-J/cm^2^ picosecond laser treatment.

### Microlens array-type 532-nm picosecond-domain laser-induced tissue reactions

**Figure 4:**
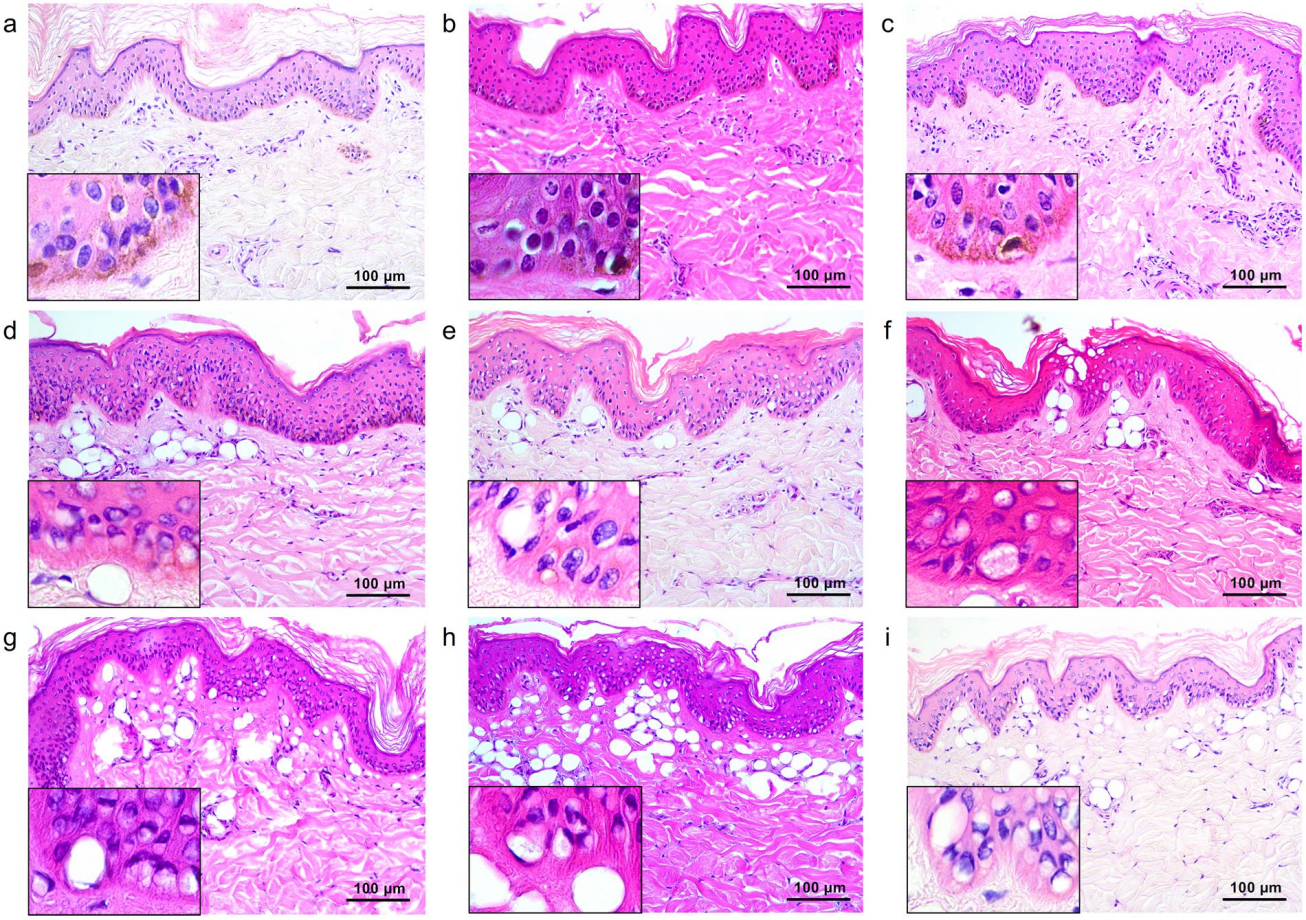
Tissue reactions after 532-nm microlens array (MLA)-type picosecond-domain laser treatment. Single pulses of MLA-type, 532-nm picosecond laser treatments at a spot size of 6 mm and fluences of (**a**–**c**) 0.04 J/cm^2^, (**d**–**f**) 0.5 J/cm^2^, and (**g**–**i**) 1.0 J/cm^2^. The distance settings between microlens and the surface of the skin were (**a**,**d**,**g**) step 1 (31 mm), (**b**,**e**,**h**) step 2 (33 mm), and (**c**,**f**,**i**) step 3 (48 mm). H&E stain, original magnification x200.

Immediately after picosecond laser treatment at a wavelength of 532 nm using an MLA-type handpiece, the laser fluence of 0.04 J/cm^2^, and the distance step of 1 over one pass generated minimal perinuclear vacuolization in epidermal cells with mildly dilated DMVCs (Fig. 4a). When increasing the distance step setting to 2 or 3, a slightly greater number of vacuolated keratinocytes were seen in the higher layers of the epidermis, compared to step 1 (Fig. 4b,c).

Skin treated at a fluence of 0.5 J/cm^2^ exhibited fractionated zones of tissue injury that were composed of signet ring-like keratinocytes and melanocytes, along with dilated DMVCs (Fig. 4d). The maximum depth of laser-induced dilated DMVCs therein was estimated at 187.0 ± 25.7 µm. The 1.0 J/cm^2^-fluenced treatment also demonstrated fractionated, but deeper and wider, zones of laser-induced tissue injury, compared to the fluence of 0.5 J/cm^2^ (Fig. 4g). The maximum depth of laser-induced dilated DMVCs was estimated at 269.2 ± 58.8 µm. Therein, the maximum depth of tissue reaction was significantly correlated with the power density (*R* = 0.989; *P* < 0.001). Moreover, the distance steps of 2 and 3 at 0.5 J/cm^2^- and 1.0 J/cm^2^-fluenced settings generated wider zones of epidermal reactions, compared to step 1 (Fig. 4e,f,h,i). Delivering an additional two passes at each setting markedly accentuated laser-induced tissue reactions in wider areas of the epidermis and dermis, compared to single pulse treatment (data not shown).

### Microlens array-type 1,064-nm picosecond-domain laser-induced tissue reaction

**Figure 5:**
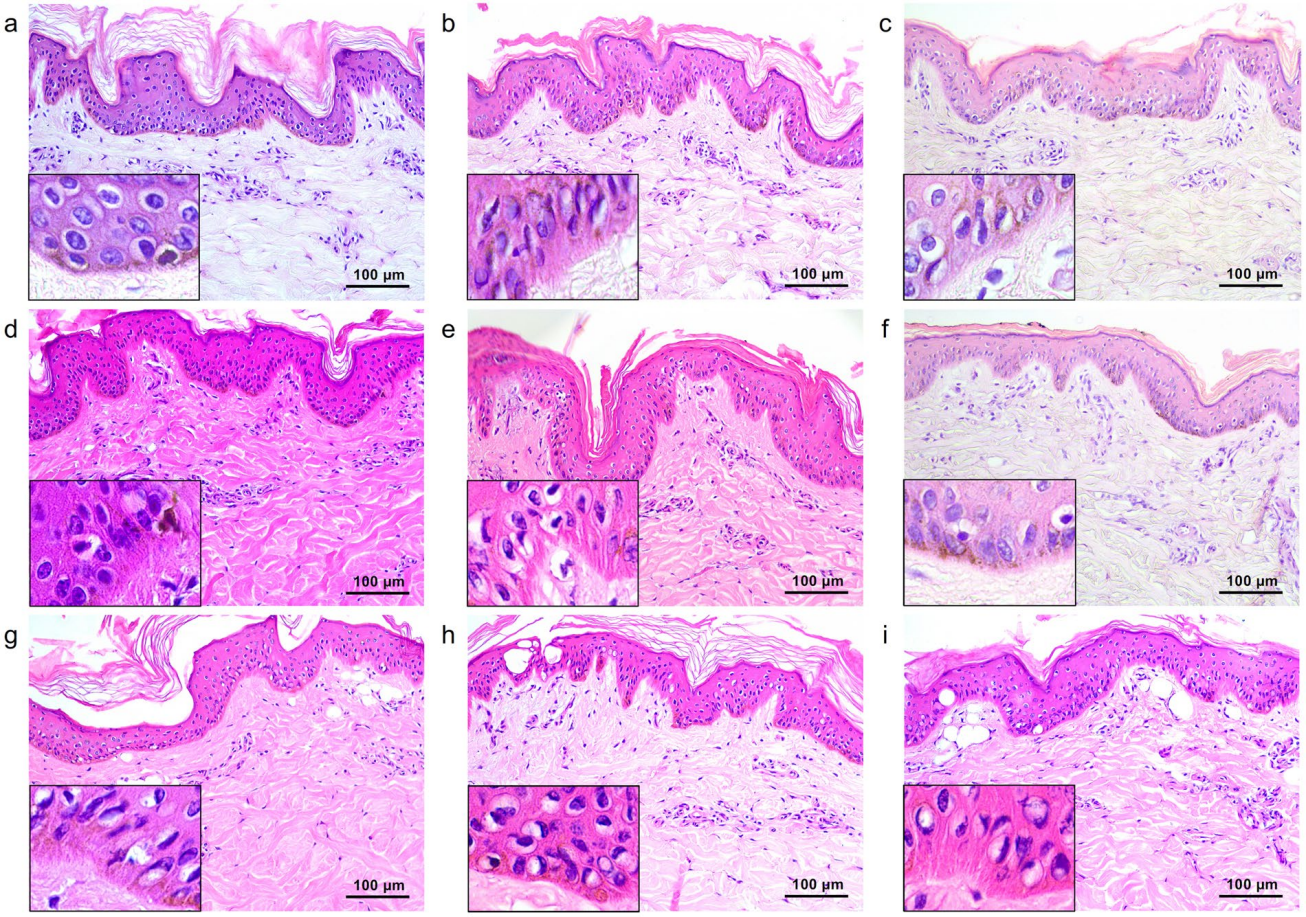
Tissue reactions after 1,064-nm MLA-type picosecond-domain laser treatment. Single pulses of MLA-type 1,064-nm picosecond laser treatments at a spot size of 7 mm and fluences of (**a**–**c**) 0.13 J/cm^2^, (**d**–**f**) 0.5 J/cm^2^, and (**g**–**i**) 1.9 J/cm^2^. The distance settings between the microlens and the surface of the skin were (**a,d,g**) step 1, (**b,e,h**) step 2, and (**c,f,i**) step 3. H&E stain, original magnification x200.

A single pass of MLA-type 1,064-nm treatment at a fluence of 0.13 J/cm^2^ and at distance step 1 generated mild perinuclear vacuolization in epidermal cells, with decreased melanin pigments (Fig. 5a). More remarkable tissue reactions were found in the upper part of the epidermis by regulating distance step from 1 to 2; step 3 exhibited similar histologic features with those at step 1 (Fig. 5b,c). Furthermore, a fluence of 0.5 J/cm^2^ generated similar patterns and degrees of laser-induced tissue reactions in both the epidermis and dermis, compared to treatment at 0.13 J/cm^2^ (Fig. 5d–f). Nonetheless, DMVCs at 0.13 J/cm^2^- and 0.5 J/cm^2^-fluence settings were unremarkable or only mildly dilated.

Laser treatment with all distance step settings at 1.9 J/cm^2^ generated fractionated areas of vacuolated, signet ring-like epidermal cells and surrounding areas of mildly to moderately vacuolated cells, with notable decreases in melanin pigments (Fig. 5g–i). Remarkable vacuolar changes in DMVCs were found, although they were limited to only the uppermost papillary dermis (estimated depth, 144.7 ± 25.8 µm). Therein, the maximum depth of tissue reaction was significantly correlated with the power density (*R* = 0.993; *P* < 0.001), and the maximum depth of tissue reaction was significantly deeper in the wavelength of 532 nm, compared to that of 1,064 nm (*P* = 0.006). Also, differences in the size of epidermal giant vacuoles between the wavelengths of 532 nm and 1,064 nm were significant (*P* > 0.05). Delivering two additional passes, MLA fractionated laser-induced tissue reactions were accentuated without excessive tissue injury (e.g., coagulation necrosis and disintegration of the skin integrity) (data not shown).

A linear mixed model analysis was additionally performed to evaluate the effects of beam modes and wavelengths on the maximum depth of laser-induced microscopic tissue reaction. In doing so, statistically significant differences in the maximum depth of tissue reaction were found between flat-top beam and MLA-type beams (*P* = 0.002) and between the wavelengths of 532 nm and 1,064 nm (*P* = 0.003). No significant interaction between beam modes and wavelengths (beam mode x wavelength), however, were noted in regards to the maximum depth of tissue reaction (*P* > 0.05).

### Horizontal sections of MLA-type laser-induced tissue reactions

**Figure 6:**
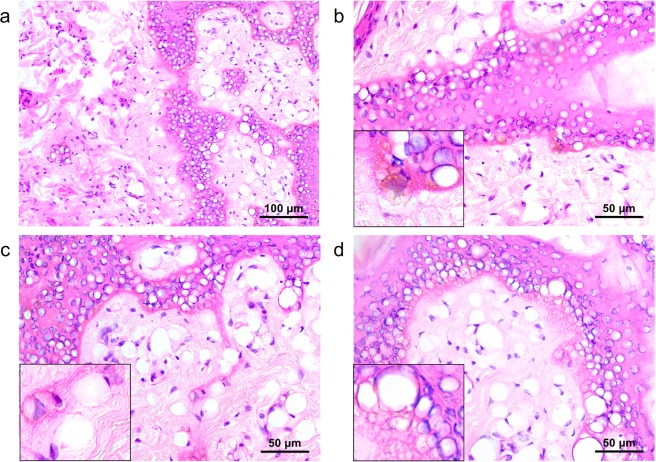
Horizontal sections of the *ex vivo* pigmented micropig skin. (**a**) A single pulse of MLA-type 1,064-nm picosecond laser treatment at the fluence of 1.0 J/cm^2^ generated vacuolated, signet ring-like epidermal cells along the rete ridges and numerous dermal components with vacuolar changes with or without nuclei. (**b**) Reduced pigment components in the basal epidermis and protrusion of vacuolated epidermal cells into the papillary dermis. (**c**) Exit of epidermal vacuolated cells from the rete ridges into the peri-rete ridge papillary dermis. (**d**) Widely and homogeneously distributed vacuolated epidermal cells with remarkably decreased pigment components. (**a**–**c**) Distance step 1 and (**d**) step 3. H&E stain, (**a**) original magnification x200, (**b**–**d**) x400.

Horizontal sections of pigmented micropig skin were obtained to evaluate histologic changes in the epidermal rete ridges and peri-rete ridge dermal tissue after delivering picosecond laser treatment at a 1,064-nm wavelength, 10-mm spot size, 1.0-J/cm^2^ fluence, and the distance steps of 1 and 3 over a single pass. At distance step 1, markedly vacuolated, signet ring-like keratinocytes were found along the rete ridges (Fig. 6a). Additionally, numerous vacuoles with or without nuclei were identified in the papillary dermis (Fig. 6a). The basal epidermis exhibited decreased pigment components with numerous tiny vacuolar changes and the protrusion of vacuolated epidermal cells, including melanocytes, into the papillary dermis (Fig. 6b). The histologic features in another area implicated the exit of epidermal vacuolated cells by laser pulses from the rete ridges into the peri-rete ridge papillary dermis (Fig. 6c). At distance step 3, the amount of pigment components decreased remarkably, compared to distance step 1 (Fig. 6d**)**.

## Discussion

In the present study, we microscopically analyzed the patterns of immediate tissue reactions generated by 532- and 1,064-nm picosecond laser treatment *ex vivo* in genotype-regulated, pigmented micropig skin. Delivery of high energy at a picosecond-pulse duration using particular lens array optics generates intraepidermal vacuoles resulting from the absorption of laser energy by melanin pigments^5,8^. Accordingly, the use of genotype-regulated, pigmented micropig skin would accentuate picosecond laser-induced tissue reactions, even with the delivery of a single pulse of picosecond-domain laser energy at relatively low energy settings.

A previous study demonstrated that more and larger laser-induced vacuoles are generated in skin with a higher melanin index when using higher energy settings^8^. In our study, tissue reactions after 532- or 1,064-nm, single flat-top beam, picosecond-domain laser treatment also exhibited similar vacuolization of irradiated components that contained chromophores of melanin and hemoglobin in the epidermis and dermis. We found that the degree of vacuolization was greater with higher fluence settings and a 532-nm wavelength, presumably due to greater absorption by both hemoglobin and/or melanin, despite higher scatter loss, at this wavelength compared to 1,064 nm.

Theoretically, laser-induced tissue breakdown is initiated by the production of free electrons during the picosecond laser pulse by one of several possible mechanisms. The free electron density increases during the laser pulse to form a plasma by repetitive free electron generation^5^. This plasma is optically thick, virtually color blind, and very efficient at absorbing energy in the remaining portion of the laser pulse. The plasma energy is then transferred to the tissue via electron-molecule collisions, and the plasma quenches after the laser pulse. The energy transferred from the plasma to the tissue generates an increase in tissue and water temperature quickly enough to create cavitation bubbles with large pressure gradients that generate vacuoles^5^.

The mechanisms for the generation of free “seed” electrons that are needed to begin the laser-induced tissue breakdown is proposed to be multiphoton absorption or thermionic emission^9^. The irradiance threshold for generating seed electrons via multiphoton absorption in water is ~10^13^ W/cm^2^ and is weakly dependent on the absorption properties of the target tissue^9^. Meanwhile, thermionic emission has a lower irradiance threshold and is much more dependent on the absorption properties of the target tissue^9^. The advantages of lowering the irradiation threshold to generate laser-induced breakdown have been suggested to include reducing the risk of collateral damage and increasing the penetration depth of the laser energy^9^. In our study, the power density for the single flat-top beam and MLA-type microbeams was less than 10^13^ W/cm^2^ and elicited remarkable histologic features of laser-induced cavitation in the epidermis and dermis in our *ex vivo* pigmented micropig model. Nonetheless, further studies are needed to investigate the precise initiation mechanism of breakdown in our experimental model.

Given our observation of vacuoles together with chromophores in this study, we deemed that the initiation of the electron plasma is not a color-blind process. The mechanisms that govern this basis (i.e., power density, wavelength, and pigment absorption coefficient) determine how early in the laser pulse the plasma is initiated and therefore how much of the remaining energy is available for plasma generation. Accordingly, the location, size, and density of vacuoles should scale with the laser fluence and pigment concentration and inversely with pigment depth (to account for beam energy loss due to tissue scatter). However, it should be noted that highly absorbing plasma effectively blocks deeper laser beam penetration.

Fractional picosecond-domain lasers have been used for treating atrophic acne scars and wrinkles^6,7,10,11^. Delivery of high-energy picosecond laser using fractionated optics produces high fluence areas, which are surrounded by low fluence background areas^5^. The injury induced by the generation of intraepidermal laser-induced breakdown theoretically stimulates the production of cytokines, chemokines, and growth factors from keratinocytes for dermal remodeling^7,12,13^. In the present study, *ex vivo* micropig skin exhibited fractionated zones of laser-induced tissue reactions in the epidermis and dermis. Therein, higher fluence settings created micro-injury zones with greater tissue reactions and higher percent coverage at both 532- and 1,064-nm wavelengths. Moreover, longer distance settings between the microlens and skin regulated the depth of tissue reaction in the epidermis and dermis.

In conclusion, our microscopic findings of laser-induced tissue reactions using *ex vivo* pigmented micropig skin demonstrated that 532- and 1,064-nm picosecond-domain Nd:YAG laser treatment generates vacuolated tissue reactions in epidermal and dermal cells. MLA-type fractionated beam delivery generated fractionated zones of vacuolated tissue reactions. Furthermore, the sizes (area) and degrees of picosecond laser-induced tissue reactions could be regulated according to the wavelength, fluence, beam type, and distance between microlens and skin. Although further clinical research using *in vivo* human skin is needed to confirm our findings, we believe that our histologic investigation may help with predicting picosecond-domain laser-induced tissue reactions in dark pigmented skin lesions.

## Methods

### Laser devices

**Figure 7:**
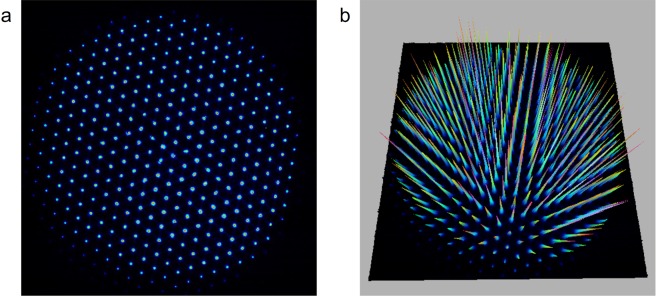
Two- and three-dimensional characterization of the MLA-type beam. The beam profiles in (**a**) two- and (**b**) three-dimensional images obtained by a complementary metal-oxide-semiconductor camera and neutral optical filters using DataRay LCMvD23 software (DataRay Inc., Redding, CA, USA). Picosecond laser treatment was performed using an MLA-type handpiece at a spot size of 10 mm, a fluence of 0.1 J/cm^2^, and a distance setting of step 1.

A picosecond-domain 532- and 1,064-nm Nd:YAG laser device (PICOPLUS; Lutronic Corp., Goyang, Korea) with a pulse duration of 450 psec was used in this study. The pulse width of this device was constant, regardless of output fluence, by adopting a master oscillator power amplifier configuration^14^. With appropriate optics, the laser energy can be delivered to target tissue as a single flat-top beam or an MLA-type beam according to therapeutic purposes (Fig. 7). The distances between the microlens and the surface of the skin can be regulated at 31 mm (step 1, microbeam size of 150 µm), 33 mm (step 2, microbeam size of 160 µm), and 48 mm (step 3, microbeam size of 300 µm). Furthermore, a nanosecond-domain 532- and 1,064-nm Nd:YAG laser device (SPECTRA XT; Lutronic Corp.) at a pulse duration of 5 nsec was used for the additional comparison experiments.

### Preparation of *ex vivo* pigmented micropig skin and laser treatment

All experimental protocols were approved by the ethics committee of the Catholic Kwandong University Institutional Animal Care and Use Committee, and the methods were carried out in accordance with the approved guidelines. Fresh back skin tissue was obtained from a Yucatan, wild-type, black female micropig (8-month old, weighing 16 kg; MK Micropig^®^; Medi Kinetics Co., Ltd., Seoul, Korea), with a genotype of *i/i* (*KIT* ^*^0101), *E*^+^*/E*^+^ (*MC*1R^*^1); the pig was sacrificed in a humane manner according to standard protocols. A total of four skin samples of 10 cm × 10 cm in size was prepared, and the skin was subsequently marked with black ink to outline 1-cm^2^ grids for each experimental setting (a total of 144 grids). Each grid was placed at least 0.5 cm from the others to minimize laser-induced photothermal and photoacoustic effects on the other areas. The temperature of *ex vivo* micropig skin was maintained between 34–36 °C on a heat plate.

Picosecond Nd:YAG laser treatments at the wavelength of 532 nm were performed separately on each grid at treatment settings with a spot size of 5.3 mm and laser fluences of 0.05 J/cm^2^, 0.1 J/cm^2^, and 1.3 J/cm^2^ over a single pass using a single flat-top beam handpiece (Table 1). Picosecond laser treatments at the wavelength of 1,064 nm were then delivered at treatment settings with a spot size of 6 mm and laser fluences of 0.18 J/cm^2^, 1.4 J/cm^2^, and 2.8 J/cm^2^ over a single pass using a single flat-top beam handpiece. Meanwhile, 532-nm, single flat-top, nanosecond Nd:YAG laser treatments were additionally performed at treatment settings with a spot size of 5.3 mm and laser fluences of 0.07 J/cm^2^, 0.1 J/cm^2^, 1.3 J/cm^2^, 1.8 J/cm^2^ over a single pass using a single flat-top beam handpiece. Additionally, 1,064-nm, single flat-top, nanosecond laser treatments were delivered with a 6-mm spot size and laser fluences of 0.36 J/cm^2^, 1.4 J/cm^2^, 2.8 J/cm^2^, and 4.0 J/cm^2^ over a single pass.

**Table 1:** Settings for picosecond-domain neodymium:yttrium-aluminum-garnet (Nd:YAG) laser treatment using a single flat-top beam on the *ex vivo* pigmented micropig skin.

Wavelength (nm)	Spot size (mm)	Fluence (J/cm^2^)	Power density (W/cm^2^)
532	5.3	0.05	1.11 × 10^8^
		0.1	2.22 × 10^8^
		1.3	2.89 × 10^9^
1,064	6	0.18	4.00 × 10^8^
		1.4	3.11 × 10^9^
		2.8	6.22 × 10^9^

Using an MLA-type handpiece, picosecond laser treatments at the wavelength of 532 nm were performed with a spot size of 6 mm and laser fluences of 0.04 J/cm^2^, 0.5 J/cm^2^, and 1.0 J/cm^2^ over a single and three passes and the distance steps of 1, 2, and 3 (Table 2). MLA-type laser treatments at the wavelength of 1,064 nm were delivered at treatment settings with a spot size of 7 mm and laser fluences of 0.13 J/cm^2^, 0.5 J/cm^2^, and 1.9 J/cm^2^ over one and three passes and the distance steps of 1, 2, and 3. To obtain horizontal micropig skin sections, MLA-type laser treatments were additionally delivered at a 1,064-nm wavelength, 10-mm spot size, and 1.0-J/cm^2^ fluence over a single pass and the distance steps of 1 and 3. All experiments were performed in triplicate.

**Table 2:** Settings for picosecond-domain Nd:YAG laser treatment using an MLA-type beam on the *ex vivo* pigmented micropig skin.

Wavelength (nm)	Spot size (mm)	Average fluence (J/cm^2^)	Average power density (W/cm^2^)	Power density of microbeam (W/cm^2^)		
				Step 1	Step 2	Step 3
532	6	0.04	8.89 × 10^7^	4.45 × 10^9^	3.91 × 10^9^	1.11 × 10^9^
		0.5	1.11 × 10^9^	5.56 × 10^10^	4.89 × 10^10^	1.39 × 10^10^
		1.0	2.22 × 10^9^	1.11 × 10^11^	9.78 × 10^10^	2.78 × 10^10^
1,064	7	0.13	2.89 × 10^8^	1.06 × 10^10^	9.33 × 10^9^	2.65 × 10^9^
		0.5	1.11 × 10^9^	4.08 × 10^10^	3.59 × 10^10^	1.02 × 10^10^
		1.9	4.22 × 10^10^	1.55 × 10^11^	1.36 × 10^11^	3.88 × 10^10^
1,064	10	1.0	2.22 × 10^9^	4.00 × 10^10^	NA	1.00 × 10^10^

NA, not applicable.

### Histological examination

Micropig tissue samples for each treatment setting were obtained 30 min after treatment, collecting the epidermis, dermis, and subcutaneous fat. The tissue samples were fixed in 10% buffered formalin and embedded in paraffin. Then, approximately 20 to 30 serial tissue sections, which were cut along the longitudinal plane at a thickness of 5 μm for each condition, were prepared and stained with hematoxylin and eosin. Additionally, 5-µm thick horizontal micropig skin sections were serially obtained and stained. The maximum depths of laser-induced tissue reactions were measured from the uppermost layer of the epidermis, except for the stratum corneum, to the deepest parts of laser-induced vacuolar tissue reactions in the dermis using Image J software (Version 1.48; National Institutes of Health, Bethesda, MD, USA).

### Statistical analysis

Values are presented as a mean ± standard deviation unless otherwise noted. Independent two-sample *t* test, chi-squared test, Fisher’s exact test, and Pearson correlation analysis were performed by parametric criteria using SAS software (Version 9.2; SAS Institute, Inc., Cay, NC, USA). Additionally, the results were analyzed via linear mixed models with Bonferroni post hoc analysis. Differences with *P* values of less than 0.05 were considered statistically significant.
