# Publisher Correction: Pattern analysis of 532- and 1,064-nm picosecond-domain laser-induced immediate tissue reactions in *ex vivo* pigmented micropig skin

**DOI:** 10.1038/s41598-019-54946-w

**Published:** 2019-12-02

**Authors:** Hee Chul Lee, James Childs, Hye Jin Chung, Jinyoung Park, Jumi Hong, Sung Bin Cho

**Affiliations:** 1 R&D Center, Lutronic Corporation, Goyang, Korea; 2 Global Center, Lutronic Corporation, Billerica, MA USA; 3 Department of Dermatology, Boston University School of Medicine, Boston, MA USA; 4 Department of Dermatology and Cutaneous Biology Research Center, International St. Mary’s Hospital, Catholic Kwandong University College of Medicine, Incheon, Korea; 5 Yonsei Seran Dermatology and Laser Clinic, Seoul, Korea

Correction to: *Scientific Reports* 10.1038/s41598-019-41021-7, published online 12 March 2019

In the original version of this Article, the author Sung Bin Cho was incorrectly indexed. This error has now been corrected.

